# Reproductive Autonomy Under Pressure: A Qualitative Exploration of Reproductive Coercion in Intimate Relationships in Canada

**DOI:** 10.1177/03616843261463188

**Published:** 2026-07-01

**Authors:** Catherine Rousseau, Sylvie Lévesque, Juliette Chevet, Carole Boulebsol

**Affiliations:** 1 Department of Psychiatry and Neurosciences, Université Laval, Québec, Canada; 2 Department of Sexology, 14845 Université du Québec à Montréal, Montréal, Canada; 3 Department of Social Work, 59310 Université du Québec en Outaouais, St-Jérôme, Canada

**Keywords:** reproductive coercion, coercive control, reproductive autonomy, Canada

## Abstract

Reproductive coercion refers to behaviors that interfere with another person's reproductive trajectory, whether by sabotaging contraceptive use, exerting pressure to become pregnant, or coercion to continue or terminate a pregnancy. Despite its profound impact, the repercussions of reproductive coercion on reproductive autonomy remain poorly documented. To address this gap, we conducted semi-structured interviews with women and non-binary people, which were transcribed and analyzed using a reflexive thematic approach. This article focused on 30 participants, aged 18 and over, who experienced reproductive coercion from a male intimate partner. Three overarching themes emerged from the analysis. These themes illustrated *the spiral,* in which control over one's reproductive health gradually erodes and becomes threatened; followed by *the chain reaction*, leading to an unplanned pregnancy; *the irreversibility*, whereby some participants experience their bodies as being placed at the service of others. A conceptual model was then developed to illustrate *the progressive narrowing of reproductive autonomy*. The findings further highlight the importance for health and social service providers of considering reproductive coercion within its broader structural context, including the cultural and social influences that shape it.

Reproductive coercion is a form of violence that specifically targets people who can become pregnant. Although the prevalence of reproductive coercion is unclear due to variations in measurement tools, the populations surveyed, and socioeconomic contexts, reported prevalence rates range from 8% to 30% (Basile et al., 2019; Falb et al., 2014; Grace & Anderson, 2018). Miller et al. (2010) initially defined reproductive coercion as a form of intimate partner violence (IPV) targeting the reproductive sphere, whereby behaviors are enacted to influence a person's reproductive trajectory. Two main categories of behaviors were originally proposed: (a) contraceptive sabotage, which includes actions aimed at damaging, hiding, or limiting access to contraceptives, and (b) pregnancy pressure, which refers to any behavior intended to force someone to become pregnant against their will. A third category has since been added and involves control over the outcome, either by pressuring someone to continue or terminate a pregnancy (Grace & Miller, 2023; Moulton et al., 2021; Silverman et al., 2011). These behaviors can be conceptualized along two dimensions: pregnancy-promoting and pregnancy-preventing behaviors (Tarzia et al., 2025). Pregnancy-promoting behaviors may include pressuring a partner to conceive, sabotaging contraception to induce pregnancy, or obstructing access to abortion. Pregnancy-preventing behaviors may encompass pressure, coercion, threats, or physical force to induce pregnancy termination (Tarzia et al., 2025). These behaviors constitute a direct threat to individuals’ reproductive autonomy.

Reproductive autonomy is understood as “having the power to decide about and control matters associated with contraceptive use, pregnancy, and childbearing” (Upadhyay et al., 2014, p. 20). Reproductive autonomy refers to the ability to decide whether or not to become a parent, use or not use contraception, and continue or terminate a pregnancy (Purdy, 2006). These choices, which should be informed and free from coercion, are shaped over time and may evolve depending on life circumstances. This concept was operationalized by Upadhyay et al. (2014) through the development of the Reproductive Autonomy Scale, which includes three subscales: freedom from coercion, communication, and decision-making. The freedom from coercion subscale assesses behaviors in which a partner interferes with prevention methods or pressures someone to become pregnant. The communication subscale assesses the presence or absence of partner support in discussions and decisions related to sexuality and reproduction. The decision-making subscale refers to who generally has the final say regarding reproductive matters, including method choice, pregnancy timing, and pregnancy outcomes. Exercising full reproductive autonomy involves the ability to revisit one's decisions and to determine if, when, and under what conditions to become pregnant, as well as whether and when to initiate or discontinue contraception. Accordingly, any behavior by an intimate partner that deliberately restricts a person's reproductive choices or autonomy constitutes a form of reproductive coercion (Grace & Anderson, 2018; Tarzia & Hegarty, 2021). Although reproductive autonomy is influenced by interpersonal dynamics, it is also impacted by sociopolitical factors that shape healthcare settings (Coleman et al., 2023).

In Canada, the healthcare system is publicly funded, providing universal access to care. In Quebec, where this study was conducted, reproductive care is considered essential healthcare, allowing for a broad range of services including contraception and abortion. Jurisprudence related to abortion ensures that women can access abortion services free of charge, within reasonable timeframes, and at any stage of pregnancy. Approximately 1 in 3 women in Canada will have an abortion during their lifetime (Government of Canada, 2024). Despite relatively broad access to reproductive healthcare and the availability of diverse resources, barriers remain. Some of these barriers include access to medication abortion and accurate information related to reproductive rights, as well as the rise of anti-abortion movements, which had previously remained relatively limited in Quebec (Action Canada for Sexual Health & Rights, 2024; Secrétariat à la condition féminine, 2024). These barriers may be more pronounced for people living in rural areas, those who speak a language other than French or English, people marginalized by race, immigrants, refugees, people living with disabilities, and others whose social positions may expose them to discrimination (Coen-Sanchez et al., 2022; Pacheco et al., 2023).

Although reproductive coercion specifically targets the reproductive trajectory, survivors have identified a range of sexual and reproductive health consequences. Notably, reproductive coercion is associated with increased risks of unplanned and unwanted pregnancies, as well as sexually transmitted infections (STIs) (Grace & Anderson, 2018; Holliday et al., 2018; Moulton et al., 2021). Mental health impacts are documented in several studies reporting associations between reproductive coercion and symptoms of depression or posttraumatic stress disorder (PTSD) (Alexander et al., 2021; McCauley et al., 2014; Price et al., 2022). People who have experienced reproductive coercion have also reported experiencing psychological distress, fear, anger, and guilt (Brodsky, 2017; Lévesque & Rousseau, 2019).

The existing research primarily approaches these consequences as outcomes rather than as dynamic processes, overlooking how they may progressively affect reproductive autonomy and how survivors resist and attempt to regain power over their experience. Some studies illustrate how the impacts of reproductive coercion persist over time and extend beyond the sphere of sexual and reproductive health, resulting in a fragile sense of safety and experience of parenthood (Pike et al., 2025). These experiences may affect people's ability to exercise their reproductive autonomy, affecting, in complex and evolving ways, the negotiation of self-perception and mental health (Wellington et al., 2025).

## Effects of Reproductive Coercion on Sexual and Reproductive Health and Well-Being

Reproductive coercion has far-reaching effects on the sexual and reproductive health of those who experience it. The risk of pregnancy, and the experience of a confirmed pregnancy resulting from reproductive coercion, may require access to services or care that enable individuals to regain some measure of reproductive autonomy. In some cases, this process involves seeking an abortion to end a pregnancy that was neither desired nor chosen (Grace & Anderson, 2018; Moore et al., 2010). Some individuals are compelled to seek abortion services in secrecy to prevent their partners from discovering their decision and obstructing access, whether by withholding financial resources, refusing transportation, or sabotaging medical appointments (Moulton et al., 2021). Reproductive coercion has also been associated with adverse maternal and fetal health outcomes. Grace et al. (2025) found that people who experienced reproductive coercion were at higher risk of delivering low-birth-weight infants and experiencing postpartum depression compared to individuals without a history of reproductive coercion or IPV. Beyond its health consequences, reproductive coercion may also shape experiences of parenthood by forcing the person to co-parent with the abusive partner, thereby creating enduring relational ties that can perpetuate control (Wellington et al., 2025), in addition to placing a financial burden related to parenthood (Spearman et al., 2025). Studies have shown that some people are reproductively coerced by their partner into carrying the pregnancy to term, confining them to an unwanted parental role (Pike et al., 2025; Willie et al., 2019). Unwanted parental roles can have complex repercussions for mental health and the parent–child relationship, which may be marked by feelings of distance, guilt, or resentment (McKenzie et al., 2024).

Reproductive coercion behaviors also harm psychological well-being more broadly. Many survivors report self-blame, questioning whether they were sufficiently clear in expressing their desire for safer sex, or blaming themselves for not being more assertive (Lévesque et al., 2020). These experiences can also lead to relational consequences, such as altered perceptions of intimacy and heightened apprehension toward new relationships. Some survivors express fear of experiencing reproductive coercion again or report a loss of trust in cisgender men, whose intentions may be perceived as potentially harmful (Lévesque et al., 2020; Rousseau et al., 2026).

## Intimate Partner Control Strategies

The relation between IPV and reproductive coercion is well documented. Several researchers have identified an overlap between reproductive coercion and other forms of violence, including physical and sexual violence as well as harassment (Tarzia et al., 2025). Psychological and verbal violence may be used to pressure someone into continuing or terminating a pregnancy. These psychological and verbally coercive strategies can include threats of ending the relationship, criticism of parenting abilities, insults related to lifestyle choices, or the use of religion or personal values to exert control and power over the partner (Bagwell-Gray et al., 2021; Boulebsol, Cousineau, et al., 2025; Lévesque et al., 2020). Studies have also shown that sexual violence may involve forcing unprotected sex to induce pregnancy, and physical violence may be used with the intent of causing a miscarriage (Lévesque et al., 2020; Muñoz et al., 2023; Suha et al., 2022). A mixed-methods study conducted in New Zealand illustrated how intimate partners may attempt to control pregnancy outcomes (Burry et al., 2020). Psychological and verbal forms of violence included partners’ denial of paternity and castigating the pregnant person (e.g., calling them a murderer should they terminate the pregnancy).

## Research Questions and Conceptual Framework

Using a reproductive autonomy framework and a feminist approach, we aimed to examine how experiences of reproductive coercion shaped participants’ reproductive autonomy, their efforts to reclaim it, and the consequences associated with their reproductive and personal trajectories. Our research questions were: How do individuals who have experienced reproductive coercion narrate their experiences and the impacts on their life and reproductive trajectories? How have they attempted to resist these behaviors to regain reproductive autonomy? Although some scholarship has identified the effects of reproductive coercion on reproductive autonomy, to our knowledge, no study has examined its progressive influence and control within individuals’ reproductive trajectories, or the ways individuals renegotiate and restore their reproductive autonomy.

As stated, we used a reproductive autonomy framework and a feminist approach to guide the interpretation of the results. The feminist approach is understood through the lens of gender power imbalances. Studies have shown that gendered power imbalances in intimate relationships can undermine individuals’ ability to control their sexual and reproductive health (Grace & Anderson, 2018; Kazmerski et al., 2015). These dynamics are often embedded within broader gendered power dynamics, where perpetrators of violence use various strategies to create conditions of vulnerability for their intimate partner, thereby exercising control (Decker et al., 2021; Maxwell et al., 2015; Thaller & Messing, 2015). These power dynamics are typically rooted in the social construct of gendered socialization processes that associate power with men (Closson et al., 2024; Lachance-Grzela et al., 2021). As a result, the bodies and reproductive potential of cisgender women and non-binary or trans people become targets of violence, undermining their freedom to make choices about their reproduction—and more broadly, their life-trajectories.

## Method

This study presents a qualitative analysis based on individual interviews with cisgender women and non-binary individuals who experienced reproductive coercion in intimate relationships. A qualitative approach allows for capturing the iterative ways in which reproductive coercion unfolds and influences reproductive autonomy over time while taking into account gendered power relations, acknowledging that reproduction is shaped by discourses embedded within a broader patriarchal context.

## Procedure

The project received ethical certification from the home university of the principal investigator (SL) (Université du Québec à Montréal, Comité institutionnel d’éthique de la recherche avec des êtres humains; Reference: 4016_e_2020). A purposive sampling strategy was used and participants had to meet the following inclusion criteria: (1) 18 years of age or older, (2) able to speak French or English, (3) a cisgender woman or a pregnancy-capable person, and (4) experienced one or more situations of reproductive coercion in the context of intimate relationships (i.e., couple, casual or short-term relationship, or dating). All individuals who experienced reproductive coercion at any point in their lives were invited to participate.

Recruitment was conducted via social media through the dissemination of a call for participation, as well as through various community organizations (sexual and reproductive health organizations and domestic violence shelters) by means of lunch-and-learn events, webinars, vignettes, and training workshops. Since the term reproductive coercion is still rarely used in everyday language, we provided examples of concrete behaviors to help participants recognize their experiences in these scenarios (e.g., a partner had ever prevented you from using a contraceptive method properly; pressured or threatened you to become pregnant; forced you to terminate or continue a pregnancy against your wishes). A 2-minute animated and narrated informational video on the various forms of reproductive coercion was also produced, in collaboration with a motion design studio, and was shared through social media in 2022. In addition to raising awareness and educating the public about reproductive coercion, the video invited individuals who identified with its content to contact the research team to take part in the study. We interviewed all individuals interested in participating who met the inclusion criteria (*n* = 33). Recruitment ended once theoretical saturation was reached. For this article, we retained only participants who experienced reproductive coercion while in a relationship with their partner (couple, casual, or short-term relationship), excluding situations that occurred in dating contexts.

Recruitment took place from May 2021 to March 2023. Most participants learned about the study through a community organization they attended. In total, 30 cisgender women and non-binary people who had experienced reproductive coercion by an intimate partner were included in the sample for the present study. The individual interviews were conducted either in person (*n* = 4) or via videoconference (*n* = 26), depending on the participant's preference. Before beginning the interview, the interviewer (CB, CR, or SL) presented the consent form and responded to any questions the participant had. The interview began once consent was formally obtained. The interviews ranged in length from 27 minutes to 2.5 hours (average duration: 1 hour and 25 minutes). After the interview, all participants were provided with a list of support resources. All interviews were audio-recorded and transcribed verbatim. Most interviews were conducted in French, except for three that were conducted in English. Selected excerpts originally in French were translated into English by the research team. All participant names used in the results section are pseudonyms randomly assigned by the research team; none correspond to the participants’ actual identities. Participants received $30 financial compensation for their participation.

## Data Collection

A semi-structured interview guide was developed to address various aspects related to reproductive coercion. The interview guide was developed based on a review of the extant literature, with the aim of identifying areas with a dearth of knowledge. The interview questions explored the relational context in which reproductive coercion occurred, issues and events related to contraception and reproduction, and the consequences experienced following reproductive coercion. Questions included the negotiation of contraception (e.g., “How would you describe the negotiation of contraception with this partner?”), as well as other forms of violence that may have been present in the relationship (e.g., “What did the situations look like in which your partner tried to control or influence your reproductive choices?”). Consequences were explored in multiple ways, particularly in relation to reproductive autonomy (e.g., “What impact have these behaviors had on your contraceptive and reproductive choices?”). One question focused on how the participant reacted in that moment (immediate reactions and emotions), as well as on the long-term impacts of these experiences. A follow-up question was included to explore the consequences of reproductive coercion on participants’ perceptions of intimate relationships, their socio-professional and academic trajectories, and their overall health (e.g., “What has been the impact on your well-being, including individual, relational, academic, and professional repercussions*?*”).

The sociodemographic questionnaire included questions documenting participants’ age, sexual orientation, gender identity, race, religious affiliation, and other characteristics. With participants’ consent, this information was used to explore how social positioning may have influenced their experiences of reproductive coercion. When relevant, we asked more specific follow-up questions, such as: “In your view, how did being in a situation of financial precarity influence how your partner perceived or behaved toward you?”

To examine the dynamic consequences of reproductive coercion, we adopted two strategies. First, we explored participants’ reactions following an instance of reproductive coercion as well as the gradual establishment of control. We sought to understand how participants recognized this control and the ways they attempted to resist it or escape from it. This strategy allowed us to document whether recognition of control occurred immediately or later, and what facilitated or hindered the recognition of the coercive nature of these behaviors (e.g., personal triggers or reactions from one's social network). Second, we examined participants’ resistance strategies and their attempts to mitigate the consequences of the violence. More broadly, our analysis focused on how these dynamics of control evolved over time, placing participants’ resistance at its center.

## Data Analysis and Reflexivity

A reflexive thematic analysis (RTA) was conducted following Braun and Clarke's (2012, 2019) approach, using NVivo 12 software (QSR International Pty Ltd). First, all verbatim transcripts were meticulously read in their entirety. Coding was then carried out using an inductive approach that remained closely aligned with the participants’ narratives. We then generated initial themes using participants’ own wording to adopt an inductive approach. These themes were subsequently reviewed and reworked collaboratively by members of the research team (CB, CR, SL). Following these revisions, the themes were further refined and named in a more interpretive manner to highlight the progressive nature of reproductive coercion on reproductive autonomy, as well as participants’ strategies to reclaim that autonomy. Themes were developed to highlight shared understandings based on the experiences described. RTA allows for the development of themes that capture what lies at the core of participants’ accounts. Data were analyzed through the reproductive autonomy framework and a feminist approach focused on gendered power imbalances within intimate relationships (Decker et al., 2021). Illustrative excerpts were then selected to best represent each theme.

The RTA approach also supports researchers in engaging in ongoing reflexive practice by documenting reflections at different stages of the research process: after each interview, during transcript review, while developing the coding structure, and during theme construction (Braun & Clarke, 2019). This process enables the research team to reflect on and engage with the data continuously. SL, the project's principal investigator, is trained in sexology and health promotion, and has been working on reproductive coercion and IPV for more than 15 years. At the time of data analysis, CB and CR were doctoral students in applied human sciences and population health, respectively, while JC was a master's student in sexology, responsible for the initial analysis. SL, CB, and CR are experienced qualitative researchers and interviewers and ground their practice in a feminist approach attentive to participants’ well-being.

Additionally, from a feminist perspective, and as recommended in the qualitative research literature (Attia & Edge, 2017; Haider, 2022), the research team engaged in ongoing reflexive practices, acknowledging how their disciplinary backgrounds, gender identities, ethnicities, and professional experiences may have shaped the co-construction of meaning with participants. We are aware that our identities may at times have led participants to perceive us, rightly or wrongly, as belonging to the same social group as themselves or as external to it. Being perceived as an insider or outsider can influence what is shared during data collection and the degree to which it is shared. For some participants, sharing cultural references with the interviewer, and knowing that these references were understood, appeared to foster greater trust during the interview process. Data analysis also led us to reflect on what constitutes a “good” excerpt and why some participants were cited more frequently than others. The findings were also discussed collaboratively within the research team to ensure they adequately reflected the diversity of experiences shared, while remaining attentive to how the experiences of marginalized participants were presented to preserve the necessary nuances. Team meetings and supervisory sessions served as key spaces for critically examining these positionalities and their influence on the research process.

## Participants

The sample included 28 cisgender women and 2 non-binary individuals (see Table 1).

**Table 1.** Participants’ Sociodemographic Characteristics.

	Total (*n* = 30)
Age	
** **18–25	4
** **26–35	12
** **36–45	13
** **46–55	0
** **55 years and over	1
Gender identity	
** **Woman	28
** **Non binary	2
Sexual orientation	
** **Heterosexual	21
** **Bisexual	4
** **Pansexual	4
** **Questioning	1
Place of birth	
** **Canada	20
** **United States and Mexico	2
** **Latin America	1
** **Europe	1
** **North Africa	1
** **West Africa	2
** **Central Africa	1
** **South Asia	2
Marginalized by race	
** **Yes	9
** **No	21
Religion	
** **None	19
** **Muslim	4
** **Catholic	2
** **Buddhist	1
** **Jewish	1
** **Other (spirituality)	3
Economic situation	
** **Financially comfortable	5
** **Sufficient income	13
** **Insufficient income	7
** **Living in poverty	5

Of the 30 participants, 23 reported experiencing reproductive coercion in the context of an intimate relationship marked by IPV. Nine participants described situations occurring with a casual or short-term partner in a relationship marked or not by IPV. The behaviors documented in this sample aimed to induce a pregnancy, ensure its continuation, or force its termination. No cases of pressure to not become pregnant were reported. All the perpetrators of reproductive coercion were cisgender men.

It is worth noting that these consequences, like reproductive coercion itself, must be understood across a diversity of contexts, including both migratory and non-migratory settings. Similarly, limited experience, young age, and financial precarity are among the factors that shaped the conditions under which the consequences presented below unfolded. These influences have been examined in greater detail elsewhere (Boulebsol, Lévesque, et al., 2025).

## Results

We identified three key themes characterized by control strategies that undermine the reproductive autonomy of cisgender women and non-binary people in intimate relationships marked by reproductive coercion, along with their consequences for decision-making and well-being. The first theme, *the spiral*, which includes three subthemes, involved initially subtle forms of control that gradually became more explicit. The second theme, *the chain reaction*, which encompasses three subthemes, refers to how reproductive coercion led to consequences for sexual and reproductive health, including unintended pregnancies. The third theme, *the irreversibility*, characterized how reproductive coercion behaviors left lasting marks on participants’ life trajectories. The fourth theme, *the progressive narrowing of reproductive autonomy*, served as both the underlying thread and a synthesis of the other themes; that is, as a conceptual model illustrating the different consequences for reproductive autonomy.

## The Spiral

Initially, reproductive autonomy was gradually undermined by partners’ subtle coercive behaviors, but these behaviors became increasingly explicit over time. The consequences reported in this initial theme included anxiety, as participants who engaged in unprotected sexual activity were unable to enjoy sex due to concerns about pregnancy or STIs. These situations progressively limited participants’ ability to exercise their reproductive autonomy.

### Eroded Control: Negotiating Contraceptive and Reproductive Decisions

Participants reported tense negotiations with their partners regarding contraceptive use or prophylactic methods. Many described their partners’ resistance to using condoms, particularly during the ovulation phase, and their insistence on relying on withdrawal before ejaculation. Several participants expressed heightened concern about experiencing an unintended pregnancy and the risk of contracting STIs. Mélanie shared her unsuccessful attempt to convince her partner to use condoms by expressing her discomfort with the withdrawal method:

It was really during a time when he would say, “You know, there's no problem. There's no risk*.*” I remember we had already talked about it, because I was like, “You know, I’m not comfortable with that, I’m really… yeah. I’m not comfortable with that, and I’m especially not comfortable with the fact that the decision you’re making is one that *I* bear the consequences of, not you.” (Mélanie, 37 years old, cisgender woman)

Negotiation became impossible as their partner imposed their decision not to use condoms, which was the method preferred by some participants. Partners’ insistence on using the withdrawal method jeopardized the participants’ sexual and reproductive health and caused significant stress: “We stopped using condoms fairly quickly because he made it clear he would always pull out in time. So, every time, it was just stressful for me” (Caroline, 40 years old, cisgender woman).

Some participants spoke about the burden of repeatedly reminding their partner of the need for protected sex:

I find it draining and… I don’t know how… it's exhausting to always have to ask again and again, because at a certain point, it's hardly enjoyable to have sexual partners if you always have to fight for them to use a condom. (Karine, 22 years old, cisgender woman)

For some, the partner's persistent resistance to condom use resulted in unintended pregnancies. Chantal described how her partner failed to withdraw, despite claiming he would:

So, well, we had sex, of course, and he didn’t want to wear a condom. I said, “Yes, wear one,” and he said, “No.” I insisted a bit. Then he said, “Don’t worry, I’ll pull out when I ejaculate.” But that's not how it works, right? So, I got pregnant. (Chantal, 59 years old, cisgender woman)

Beyond resistance to condom use, participants also reported that their partners resisted other contraceptive methods, such as intrauterine devices (IUD), criticizing the degree of control it provided over reproductive planning. One participant scheduled an appointment to have an IUD inserted, but her partner objected when he found out: “I remember we had the conversation, and he told me, I’ll probably remember this forever*, ‘*I’m not sure I agree with the idea of an IUD because it takes away the magic of life’” (Chiara, 32 years old, cisgender woman). In this situation, the partner was aware of the risk for pregnancy and welcomed it. He opposed her efforts to prevent an unintended pregnancy by invoking guilt and value-based arguments.

Although some participants tried to find alternative means of protection, others had a method of contraception imposed on them without their consent. This was the case for a non-binary participant whose significantly older partner refused to use condoms, claiming they caused erectile difficulties. He pressured the participant to start using the birth control pill instead and eventually forced them to take it, even though it was not the participant's preferred method: “There was a part of my agency that just didn’t exist” (Manu, 29 years old, non-binary).

The imposition of unprotected sex (without a condom or other contraceptives) or a less effective contraceptive method (withdrawal before ejaculation) constituted a form of coercion that generated stress, concerns, and fears related to pregnancy and STIs, and exhaustion, which undermined reproductive autonomy. Participants described their partners’ pressure to avoid using contraception as a barrier to exercising their reproductive choices and protecting themselves against unintended pregnancy.

### Threatened Control: Anticipating and Acting to Preserve Reproductive Autonomy

Partners’ resistance to having protected sex led participants to remain vigilant and to implement strategies aimed at maintaining or regaining control over their reproductive autonomy. These attempts to regain control required constant awareness of the risks posed to their sexual and reproductive health and anticipation of their partners’ behaviors. Some participants reported obtaining emergency oral contraception following either unprotected sex or contraceptive sabotage. These situations stemmed from their partners’ refusal to use condoms or withdraw before ejaculation. Some participants also described cases in which their partners removed the condom without their consent or knowledge during intercourse (non-consensual condom removal).

In addition to turning to other contraceptive methods, some participants attempted to modify their sexual practices, particularly during the ovulation period, to reduce their risk of pregnancy. Yasmine reported agreeing to sexual practices she disliked and that caused her injuries and physical pain in order to avoid pregnancy:

I no longer trusted him because I knew he wanted to get me pregnant. He wanted a child. […] One of the things he liked to do against my will and in a brutal way was anal sex. Without lubrication, and very roughly, which hurt me. But in my mind, I told myself that this was an option to avoid getting pregnant during ovulation. So, I was willing to accept it or even suggest it, even though I knew I would end up injured, because I thought that way I wouldn’t risk getting pregnant. (Yasmine, 40 years old, cisgender woman)

As a result of their partners’ coercive behaviors and boundary violations, participants reported hypervigilance and modifying contraception and sexual practices to reduce the risk of unintended pregnancy. These efforts sought to re-establish some degree of reproductive autonomy and to avoid consequences that could change their life trajectories, such as an unintended pregnancy.

### Seized Control: Feeling Trapped by the Constant Risk of Pregnancy

Participants progressively identified their partners’ controlling strategies as deliberate strategies aimed at causing pregnancy. Despite efforts deployed by participants to avoid exposure to unprotected sex that could result in pregnancy, most of them reported feeling trapped by the constant threat of it. In several cases, the partner intentionally created situations that placed participants at risk of becoming pregnant, making it difficult—if not impossible—for them to preserve their reproductive autonomy. Participants described a climate of fear and the presence of physical and sexual violence that constrained the exercise of reproductive autonomy. Participants also reported economic constraints by not having enough money to access, for example, emergency contraception to regain some degree of reproductive autonomy. These forms of violence acted as a tightening constraint on participants’ autonomy and increased the risk of pregnancy.

One participant shared that her partner threw away her birth control pills to intentionally cause a pregnancy: “So yeah, for the first [pregnancy], he threw away my birth control pills. He just took them and tossed them in the trash, like*,* ‘I want to get you pregnant’” (Stéphanie, 35 years old, cisgender woman). Another participant recounted how her partner forced unprotected sex during ovulation, despite their prior discussions about the importance of being cautious during that period: “We had sex, and he knew I was ovulating. We had talked about it a lot, how he had to be extra careful when I was ovulating. And…he ejaculated inside me while I was ovulating. On purpose” (Yasmine, 40 years old, cisgender woman). In some instances, participants’ efforts to negotiate contraception were met with frustration or the escalation of violence. Jessica described how her partner responded violently after she suggested using a condom, forcing unprotected vaginal intercourse and manipulating her body in ways he believed would enhance fertilization:

And then, like…he just looks at me…kind of weirdly, and then he starts again, slowly. And then he gets rough. He gets rough, and he doesn’t change position, nothing. And he makes sure to come inside me […] And I’m just like: “Okay. I’m screwed”. (Jessica, 39 years old, cisgender woman)

These instances of sexual violence severely limited, if not entirely eliminated, participants’ control over their reproductive autonomy. Some participants also reported that their partners refused to contribute financially to the cost of emergency contraception, which, in a context of financial insecurity, represented a significant barrier to preventing unintended pregnancy:

“You didn’t have a condom, you refused to use the one I brought, and again you ejaculated inside me after I told you I wasn’t on birth control.” And then the next day I said: “Well, I don’t have the money right now, $25, to get Plan B. What are you going to do?” He did nothing. I never heard from him again. (Chiara, 32 years old, cisgender woman)

This fear was heightened by an awareness of how pregnancy in a violent relationship could additionally affect one's well-being. For instance, when discussing pregnancy, Léa detailed the importance of timing and the right partner: “I want to have a child, but definitely not now. And at that moment, even less. And with that person, even less” (Léa, 24 years old, cisgender woman). Some feared that pregnancy would trap them in a violent relationship, making it even more difficult to leave:

For two weeks, I was terrified. I was scared I was pregnant, because I thought: I’ll be trapped. I’ll be trapped. If I’m pregnant, I won’t be able to get an abortion. So, I’ll be stuck in this relationship, this situation, for who knows how long? (Yasmine, 40 years old, cisgender woman)

Participants felt they were left with no options to escape the risk of pregnancy. The fear of becoming pregnant had substantial consequences not only for their sexual and reproductive health, but also for their physical and mental well-being. Their reproductive potential was exploited by their intimate partners, who imposed pregnancy by systematically eliminating opportunities to avoid it.

### The Chain Reaction: Becoming Pregnant…

In some cases, behaviors of reproductive coercion resulted in pregnancy. Although some participants were able to access abortion services as they wished, others had it forced upon them by their partner. In other cases, participants were forced to carry a pregnancy to term against their will. In all these situations, participants’ reproductive autonomy was compromised, with profound impacts on their sense of self and their perception of the intimate partner, the pregnancy, and the child.

### …and Being Able to Terminate the Pregnancy

Some participants became pregnant as a result of contraceptive sabotage and were able to carry out their decision to have an abortion. In such situations, participants retained the option of terminating the pregnancy, but nonetheless expressed a sense of lost control over their reproductive trajectory, as the timing and circumstances of the pregnancy were not of their choosing. Although these participants were able to have an abortion in accordance with their wishes, some recounted feelings of guilt and fear, in addition to harassment from their partner. They described experiencing both the physical and emotional effects of early pregnancy symptoms, the distress of discovering the pregnancy, and the urgency of deciding whether to continue or terminate it.

One participant described having two closely spaced abortions due to repeated non-consensual condom removal by her partner: “He did the same thing again and I got pregnant a second time. And I had to get another abortion, like two in a row…basically two months in a row” (Laurie, 36 years old, cisgender woman). These acts of reproductive coercion led her to undergo a medical procedure she would have preferred to avoid through safer sexual practices.

Even when abortion was the participant's decision, it was sometimes accompanied by psychological violence from the partner. Gabrielle described how her decision to terminate the pregnancy was deliberate, though painful, in a context where her partner opposed the procedure and accused her of “killing their potential child”:

And when I told him I wanted to get an abortion [brief silence], this time he said, “No. It's the brother or sister of [nickname of her son].” […] I told him, “No. I can’t. I can’t. I can’t.” A month later, things really started to go downhill with [name of ex-partner]…That's when he really said, like…“You killed your daughter, you know.” [brief silence] “You killed the daughter you wanted.” (Gabrielle, 31 years old, cisgender woman)

These words continued to haunt the participant for years, triggering immense guilt despite her certainty that it had been the right decision for her well-being, especially since she had been a minor at the time. This strategy of emotional manipulation was also reported by another participant: “He kept telling me that if I got an abortion, I’d be killing a human being, and he really tried to make me feel bad about it” (Melissa, 25 years old, cisgender woman). Even after the end of the relationship, the fear of being confronted again by her ex-partner and being blamed for her decision remained present:

Of course there's anger and sadness, but there's also fear. Fear of him. I’m still afraid he’ll find me, even today. I’m still afraid he’ll blame me again for having had the abortion, because it would be just like him to do that. (Melissa, 25 years old, cisgender woman)

Although the decision to terminate the pregnancy allowed these participants to reclaim a degree of reproductive autonomy, it was also accompanied by persistent doubts, guilt, and fear that could linger long after the incidents of reproductive coercion.

### …and Being Forced to Terminate the Pregnancy

Although some participants chose abortion to regain reproductive autonomy and a sense of control over their life, others were pressured by their intimate partner to terminate a pregnancy. These participants wished to carry the pregnancy to term, even though it had not been initially planned. One participant, Noor (34 years old, cisgender woman), recounted that, upon disclosing her pregnancy to her husband, he immediately rejected the idea and coerced her into having an abortion against her will. He used physical violence to frighten her by smothering her with a pillow. Another participant recalled how her partner manipulated her values to induce guilt about her desire to continue the pregnancy:

He would call me…in the middle of the night, in the morning, every day, we’d meet and the topic was always: “Please have an abortion, have an abortion, abortion*…*” […] He started telling me I wasn’t a feminist because I didn’t want to have the abortion, that it wasn’t consistent with what I had said, that I wasn’t a feminist at all. He started using coercion around feminism and the church. (Mathilda, 45 years old, cisgender woman)

Some participants described how their partners shifted erratically in their stance. At first, they encouraged the pregnancy, only to later pressure them into having an abortion. For example, in the context of a migration process, one partner initially pressured her to become pregnant, believing that having a child would accelerate administrative procedures. Upon learning that this was not the case, he completely reversed his position and demanded that she terminate the pregnancy. This inconsistency created confusion and distress, leaving the participant feeling trapped. Likewise, Sabrina shared that her pregnancy occurred after her partner previously encouraged her to remove her IUD, monitored her menstrual cycle, calculated her ovulation date, and ejaculated inside her. Yet, once she became pregnant, he pressured her to abort:

There was so much going on, I had trouble thinking clearly. It felt like being in a storm, you can’t see straight. That's how I felt. I felt kind of trapped…like I was the one faced with the choice, you know? And I felt betrayed too. I’d say it was mostly a sense of betrayal. Because it's like he did everything he could to make it happen, to get me pregnant. And then once I was […] [he] acted like it was a mistake […]. (Sabrina, 30 years old, cisgender woman)

These manipulative acts caused emotional destabilization. Intimate partners targeted participants’ belief systems and values in an attempt to influence their decisions about whether to continue the pregnancy. Underlying these strategies were guilt-inducing strategies aimed at manipulating participants into terminating their pregnancies.

### …and Being Forced to Carry the Pregnancy to Term

In other cases, pregnancies were carried to term following manipulative strategies by the partner, which rendered the option of abortion inaccessible. For example, participants reported that their partners sought to isolate them from their social networks and disrupt their educational and professional trajectories. Participants also described physical barriers to accessing healthcare services, resulting in unmet care needs. Collectively, these situations had significant repercussions for participants’ mental health and self-perception.

Nadia shared how her partner manipulated her social environment to only expose her to traditional family models, often with multiple children:

What he did, because of the age difference and his greater life experience, was bring me around his friends who had babies at the time. It's like he chose my friends for me – well, the wives of his friends, in his circle. […] So I’d go over, I’d see the little babies, and I’d think, “Oh, it's cute, such a beautiful family,” so it's like he was showing me an example. It was indirect manipulation. His friends were well chosen, carefully selected, from the [religious] community, he brought me to the [place of worship], things like that. (Nadia, 37 years old, cisgender woman)

This partner also raped her on multiple occasions in an attempt to cause pregnancy, ultimately succeeding by also preventing her from using contraception, claiming it would negatively affect the quality of her breast milk. Each subsequent pregnancy further entrenched her in the relationship: “It was all planned, like a Machiavellian plan, that's what I call it – to completely block me, so I wouldn’t have a career, no money. The burden is heavy*”* (Nadia, 37 years old, cisgender woman). Although she considered abortion and sought information about it, she ultimately chose not to proceed, primarily for religious reasons.

Other perpetrators of reproductive coercion ensured that the unwanted pregnancy would be carried to term by discouraging abortion through guilt-inducing comments or by physically blocking access to services. Nathalie described how her partner, in alliance with her mother (who wanted to become a grandmother), manipulated and pressured her to cancel her abortion:

I gave in because it gave me the love I needed. Giving them a child brought me the love I craved. So, I gave in because I was afraid of what would happen if I didn’t keep it. I was afraid of the consequences of my own decision. And since they said I was selfish, that I was only thinking about myself, that really hurt my self-esteem. (Nathalie, 42 years old, cisgender woman)

The fear of losing her relationship with her mother and her partner by not giving them a child, combined with the guilt they made her feel, placed her in a situation where she perceived no real choice but to continue the pregnancy. Ultimately, she sacrificed her reproductive autonomy to preserve relationships with her partner and her mother. Another participant, Sophie, recounted how her partner followed her to the abortion clinic and created a scene, loudly objecting to the procedure. The disruption caused so much distress that the clinic and the participant decided to postpone the intervention. The abortion was never rescheduled and Sophie carried her pregnancy to term.

According to some participants, forced pregnancy was intended to create a long-term relational tie between them and their partner. Sophie shared how her partner saw a child as a way to ensure she would always owe him something:

I feel like when he said he wanted a child, it was like a life raft for him: If I had a child with him, I would owe him something… respect, or some kind of emotional bond. He thought we’d stay together, that I wouldn’t leave him, and that I’d remain submissive to him because we had a child together. (Sophie, 34 years old, cisgender woman)

Intimate partners employed a wide range of coercive strategies—threats, insults, manipulation, and physical obstruction—to enforce the continuation of a pregnancy. As a result, participants were forced into parenthood against their will.

## The Irreversibility: Dispossessed of One's Body in the Service of Others

Reproductive coercion created permanent life circumstances for many participants, whether through the arrival of a child or the long-term consequences they experienced. The sense of irreversibility ran through their stories, revealing how bodily autonomy and self-determination were eroded in ways that profoundly altered participants’ life trajectories. Participants described having been objectified, used, dehumanized, and constrained into a life they did not choose. These experiences contributed to difficulties integrating a parental identity and developing a bond with their child.

Several participants spoke of feeling used for their reproductive capacity, with no regard for their individuality or subjectivity, leading to a profound sense of dehumanization. Mélanie, for example, shared that she never chose the pregnancy and recalled feeling as though she was “delivering” the child to her partner after giving birth:

[…] I gave birth, they placed my baby in my arms. And I don’t remember how long it took, but it wasn’t long before I took the baby and placed them in my partner's arms. Not because I wanted to share that moment with him, but because I felt I had to put the baby in his arms. [crying] […] It was like I was delivering his baby to him. (Mélanie, 37 years old, cisgender woman)

Another participant described feeling used at different points throughout her life by the same partner, specifically for her reproductive capacities:

What I take away from all of these situations is that I felt used. I felt objectified. […] Like I was… a means to an end. […] Like a gestational vessel [small laugh] for this person. When really, I didn’t want that. (Léa, 24 years old, cisgender woman)

Participants were thus forced into a reproductive trajectory they did not choose. Nadia, speaking of her closely spaced pregnancies, said: “It changed the course of my life. This is not the life I wanted. It's not… this is not what I signed up for” (Nadia, 37 years old, cisgender woman). A participant, who shared earlier that she had a child under pressure from both her mother and her partner, summarized the experience as follows: “I had the child for others. I gave in to their pity so I wouldn’t hurt them” (Nathalie, 42 years old, cisgender woman). At birth, she had trouble bonding with her baby, recounting that she was in shock:

It happened so fast [the birth], I went into shock, and when they wanted to hand me the baby, I didn’t want her. I rejected her. Even the nurses were saying, “Come on, it's your baby,” and I was like, “no, no.” I didn’t want her. I didn’t even want them to lay her on my chest. (Nathalie, 42 years old, cisgender woman)

These reproductive coercion situations created contexts where adapting to pregnancy, childbirth, and then the arrival of the baby became deeply complicated. The process of forming a maternal identity could also be affected:

For me, it's like this pregnancy took away… and maybe it's just in my head [crying]… it took away all my independence. […] From the moment I gave birth, I no longer had the right to be myself. From the moment I gave birth, I became a mother and only a mother. (Mélanie, 37 years old, cisgender woman)

Participants who were pushed into parenthood against their will experienced significant, enduring consequences. Reproductive coercion involved a violation of physical integrity by disregarding the reproductive decisions of participants. The consequences varied, but reproductive coercion left a lasting imprint on the lives of survivors, whether through the fear of becoming pregnant, the need to undergo abortions, or the coercion to either terminate or carry a pregnancy against their will.

## The Progressive Narrowing of Reproductive Autonomy

The consequences of reproductive coercion on reproductive autonomy (see Figure 1) ranged from pressures that temporarily destabilized participants’ life trajectories but were later mitigated (e.g., accessing abortion services) to the complete dispossession of their bodies, producing lasting consequences on life trajectories (e.g., having a child). Participants’ decision-making power varied, from a reduced ability to make choices due to coercion, to the need to implement resistance strategies to try to mitigate the consequences. This model does not necessarily represent a chronological sequence or a linear progression of reproductive coercion. Rather, it provides a visual representation of consequences that function as processes of (dis)appropriation of reproductive autonomy. The model is not intended to standardize the singularity of participants’ experiences. Rather, it offers an interpretive synthesis of recurring turning points identified across participants’ narratives. The components of reproductive autonomy, as defined by Upadhyay et al. (2014), guided the interpretation of the reported experiences and consequences.

**Figure 1.**
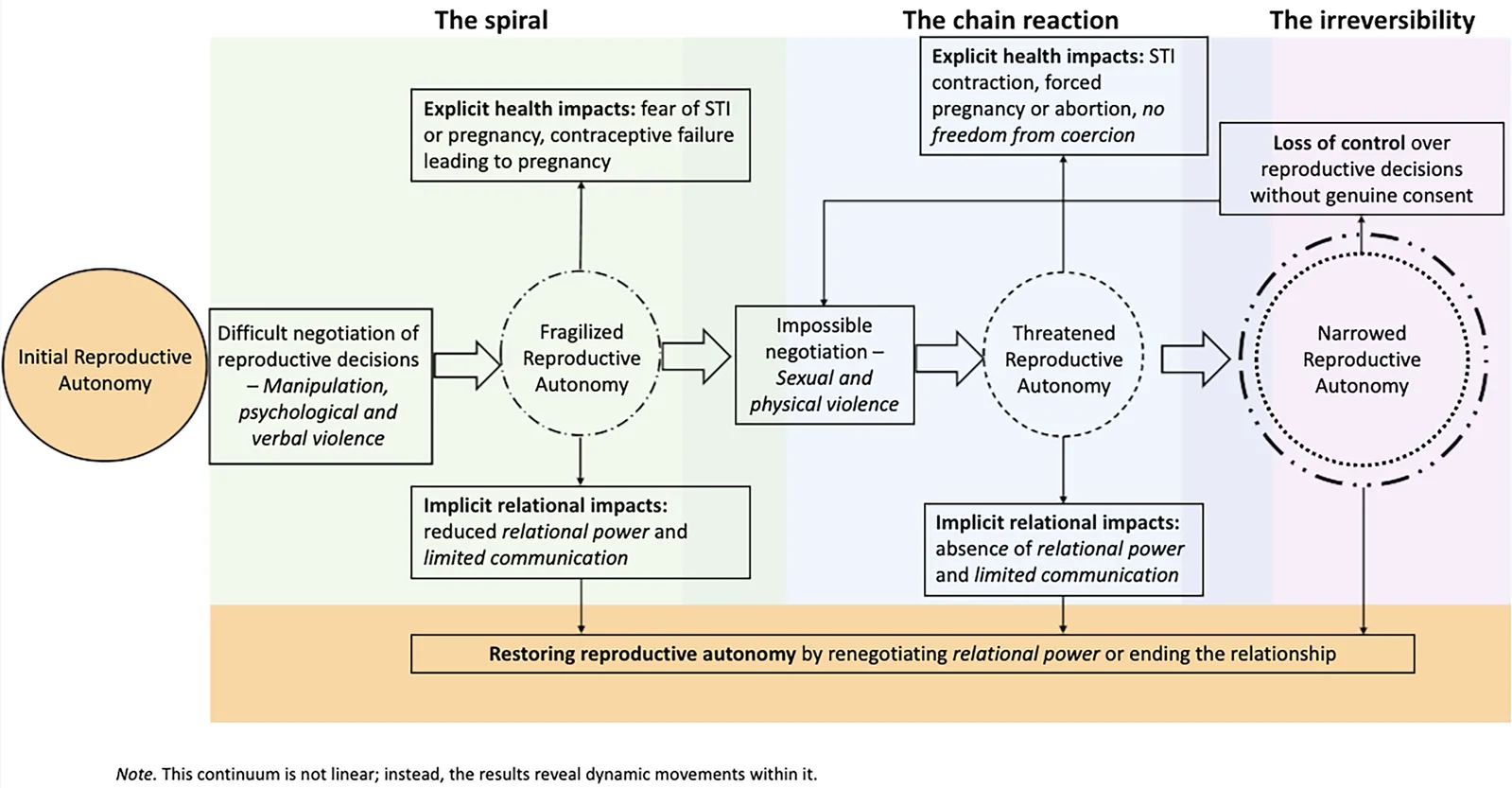
Continuum of control over reproductive decisions and the erosion of reproductive autonomy.

As illustrated in Figure 1, when reproductive autonomy was reduced, participants had to adapt to new contextual parameters. Three distinct paths emerged: regaining previous parameters of reproductive autonomy by leaving the violent relationship, attempting to renegotiate reproductive autonomy with the partner, or yielding, without consent, to the partner's control over reproductive decisions. The first path involved ending the relationship with the perpetrator of reproductive coercion, allowing participants to recover their initial level of reproductive autonomy; that is, what they had before entering the relationship. In the second path, participants adopted behaviors aimed at reclaiming their previous reproductive autonomy. For instance, taking emergency contraception after non-consensual condom removal was a way to avoid a pregnancy resulting from reproductive coercion. The third path did not necessarily involve expanding their reproductive autonomy, but rather adapting to reduced possibilities. For example, modifying sexual practices during ovulation was a resistance strategy that enabled participants to cope with a diminished reproductive autonomy. These strategies helped prevent further erosion of reproductive autonomy. These three paths were not mutually exclusive, as a person might experience all of them at different moments within the same intimate relationship marked by reproductive coercion.

## Discussion

The objective of this article was to examine the strategies through which reproductive coercion compromises or prevents the exercise of reproductive autonomy among women and non-binary individuals. We also analyzed the strategies through which participants reasserted their reproductive autonomy and the broader implications of these processes for their reproductive and personal trajectories. We developed a model of reproductive coercion consequences centered on reproductive autonomy. The findings highlighted how perpetrators used control and domination to exert control over their partner's reproductive trajectory. These results contribute novel insights by illustrating both the progressive erosion of reproductive autonomy and the ways survivors attempted to reclaim power or renegotiate its terms in the context of reproductive coercion. In this sense, this article complements a body of literature that has primarily focused on health-related impacts and dysfunctions of reproductive coercion (Grace & Anderson, 2018; Moulton et al., 2021) by shifting the analysis toward power relations and processes of (dis)empowerment.

Our findings illustrated that reproductive coercion influences reproductive autonomy, both in its actualization and in sexual and reproductive health outcomes. Reproductive coercion behaviors also act with varying intensity and frequency across the three components of the *Reproductive Autonomy Scale*: decision-making, communication, and freedom from coercion (Upadhyay et al., 2014). In our study, situations related to *decision-making* were described by participants as moments, or turning points, when contraception was negotiated or when discussions around parenthood occurred. These negotiations revealed the distribution of decision-making power within the relationship. In reproductive coercion contexts, this power quickly became unbalanced in favor of the partner. By imposing the conditions of sexual intercourse, some participants reported they had no final say over the use or non-use of contraception. These narratives aligned with prior findings on men's condom use. That is, men frequently engage in condom use resistance, sometimes through explicitly coercive behaviors (Davis et al., 2019), or by attempting to avoid condom use in various ways, such as through coerced condomless sex (Chen et al., 2024) or non-consensual condom removal (Gómez-Durán & Martin-Fumadó, 2024). These coercive strategies left little room for women and non-binary individuals to assert themselves (Brodsky, 2017; Grace et al., 2022). In this regard, participants often reported multiple forms of violence (sexual, physical, and/or psychological), thereby undermining opportunities for shared decision-making. In the figure, we used the term “relational power” to encompass both decision-making and gendered power imbalances, to more faithfully represent participants’ accounts.

These abusive contexts prevented participants from experiencing *freedom from coercion* (Upadhyay et al., 2014). Participants described their intimate partners’ behaviors as having forced them into reproductive trajectories they had not chosen. Various controlling strategies were deployed through reproductive coercion (Bagwell-Gray et al., 2021; Rowlands & Walker, 2019), including contraceptive sabotage (e.g., destroying contraceptive methods, preventing condom use), pressure to promote pregnancy (e.g., threats, blackmail, harassment), and control over pregnancy outcomes by either forcing continuation or termination of a pregnancy. Reproductive coercion progressively established control over participants, whose reproductive autonomy diminished. These behaviors were typically accompanied by psychological, verbal, physical, and sexual violence, creating an explicit dynamic of control within the intimate relationship. As Sheeran et al. (2024) observed, people who experienced reproductive coercion may report behaviors from their partners that aim to both promote and prevent pregnancy. As our findings suggested, the rules of the intimate relationship shifted over time, with the partner's grip and demands adapting, consistent with coercive control literature (Stark, 2007). These rules, often implicit and sometimes negotiated, dictated which behaviors must be followed or avoided according to the partner's expectations. Reproductive coercion behaviors were shaped by a range of controlling strategies, from subtle to explicitly violent (e.g., Côté & Lapierre, 2021). Such strategies limited participants’ reproductive autonomy, leaving them with a sense of being trapped (e.g., Wellington et al., 2025).

*Communication*, which includes both the comfort level in expressing one's boundaries to a partner and the trust that those boundaries will be respected, is difficult to explore and document in a reproductive coercion context. Indeed, assessing communication in violent dynamics may be ineffective, as sexual, physical, or psychological violence annihilates any possibility of expressing one's boundaries or trusting that they will be respected. Reproductive coercion behaviors are, by nature, acts of violence that prevent transparent communication. Many participants, upon recognizing the coercion they experienced and their partner's disregard for their contraceptive and reproductive choices, completely abandoned any expectations of healthy communication. As a result, their trust in their partner deteriorated, leading them to resist coercive behaviors in an effort to reclaim their reproductive autonomy. This resistance at times involved having an abortion without informing the partner, obtaining emergency contraception, or eventually leaving the relationship (Humphreys & Sheeran, 2025). On the other hand, some participants found themselves stuck in the relationship. Thus, *decision-making* and *communication* are interrelated: since the partner made most, if not all, decisions regarding contraception or parenthood, the possibility of expressing boundaries and trusting that they will be respected ceased to exist.

Reproductive coercion behaviors resulted in consequences that went beyond sexual and reproductive health, as participants reported a wide range of long-lasting impacts over different timeframes. Their life trajectories diverged from what they envisioned, whether due to closely spaced pregnancies, multiple abortions, or the presence of children for whom they were responsible. Some participants reported feeling dispossessed of their bodies, instrumentalized for the purposes of reproduction. This echoes the concept of body territorializing, which refers to a set of practices aimed at controlling women by specifically targeting their bodies (Gago, 2020). For example, in a study of 97 men incarcerated for IPV or femicide (Di Marco & Evans, 2025), body territorializing manifested through behaviors restricting women's movement, typically confining them to the home, as well as interfering with their reproductive autonomy. Specifically, these participants sought to control women's reproductive trajectories, including forced pregnancies through sexual assault, confiscation of contraception, or opposition to abortion. Reproduction may thus become a tool for confining pregnancy-capable people to domestic life and the intimate relationship, where their bodies are treated as a territory “conquered” by men. This finding is consistent with research by Tarzia and Hegarty (2021), who found that survivors of reproductive coercion experienced domineering behaviors aimed at controlling their bodies and reproductive capacities (i.e., being reduced to one's bodily functioning by means of having a child).

## Practical Implications

These findings highlight the importance of actively fostering reproductive autonomy among cisgender women and pregnancy-capable people. Reproductive autonomy may lead individuals to leave relationships marked by reproductive coercion or to resist its effects by attempting to mitigate risks to their reproductive health and well-being. As reproductive coercion remains poorly understood among the general population, it is important to educate individuals about what reproductive coercion behaviors can look like, helping them recognize that these behaviors constitute a form of violence within intimate relationships. Such education could also address myths suggesting that controlling behaviors are an acceptable form of “care” in intimate relationships or that one partner, because of their life experience, is uniquely legitimate in making decisions for the couple. In Quebec, mandatory sexuality education is included throughout the school curriculum, from primary through secondary education (ages 5 to 17). These programs could incorporate content on reproductive coercion to promote awareness beginning in adolescence and contribute to the prevention of this form of violence.

Relatedly, another intervention target may involve strengthening agency, which is often significantly undermined by reproductive coercion. Health and social service providers could support individuals who have experienced reproductive coercion in developing new communication frameworks that validate their boundaries and needs. Overall, these interventions would benefit from an approach that is sensitive to cultural and social influences, as the findings illustrate how values, religion, and life aspirations can be instrumentalized to further entrap individuals in abusive relationships. Reproductive coercion undermines the reproductive autonomy of survivors, but their experiences are not exclusively dyadic; they are embedded within a broader social context that may either facilitate their resistance or, conversely, hinder it. In this regard, health and social services providers have an important role in offering support to survivors. This recommendation aligns with other studies suggesting that reproductive autonomy should not be understood solely as constrained by direct interpersonal behaviors, but also as shaped by the broader structures that may influence access to services and care (de Haas et al., 2025; Saldanha et al., 2025).

## Limitations

Participants may have withheld certain aspects or emotions related to their experiences due to social desirability bias. Indeed, having had an undesired child against one's will can be associated with social taboo. Those who experienced such situations often reiterated their love for their child, even though the circumstances of the pregnancy were ones they would have preferred to avoid. Despite the efforts of the interviewers (CB, CR, SL) to create a safe, non-judgmental environment by validating participants’ experiences and explicitly acknowledging the normalcy of feeling contradictory emotions in such contexts, it is possible that some participants did not feel comfortable sharing the full range of their experiences.

Second, although we sought to explore the potential influence of social positioning on the experience of reproductive coercion as reported by the women and non-binary participants, the data allowed only a partial understanding of these intersections. The findings highlighted how certain life contexts, such as financial precarity, migration trajectories, and specific values or religious beliefs, were instrumentalized by the perpetrator of reproductive coercion to exert greater power and control over the participant. Future studies could specifically focus on these dimensions to provide a more comprehensive understanding of the control strategies employed by men within the context of reproductive coercion.

Third, some participants reported experiences of reproductive coercion that occurred several years prior; therefore, recall bias may have influenced their accounts. In spite of these limitations, this innovative study offers a qualitative analysis of reproductive coercion at the intersection of reproductive autonomy. It is relevant to focus on violations of reproductive autonomy when addressing reproductive coercion, as this form of violence limits the ability to protect or regain optimal sexual and reproductive health. These two interconnected concepts help to better understand the implications of reproductive coercion on sexual and reproductive health, and more broadly, on the life trajectories of cisgender women and pregnancy-capable people.

## Conclusion

The stories of the cisgender women and non-binary people interviewed highlight the multiple ramifications of reproductive coercion, particularly regarding the exercise of their reproductive autonomy. In addition to the risks posed to sexual and reproductive health, these experiences can permanently alter the course of women's lives, such as the unplanned arrival of a child or the experience of an (un)wanted abortion. Further empirical research is needed to better understand the consequences of reproductive coercion on reproductive autonomy and on mental well-being, including relational repercussions, and how the behaviors affect decision-making, trust, communication, and gendered power dynamics within intimate relationships.
